# Workforce Diversity Interactions and Perceptions Among Nurses in a Tertiary Maternity Facility in Qatar: A Sequential Explanatory Mixed‐Methods Study

**DOI:** 10.1155/jonm/2649393

**Published:** 2026-02-15

**Authors:** John Paul Ben T. Silang, Evalyn Abalos, Barbara Lyn A. Galvez, Theresa Guino-o, David Hali de Jesus, Hazel F. Adalin, Rana Aatif Salim Ibrahem, Jameela Syed Roshan Nuddin, Norisk M. Adalin

**Affiliations:** ^1^ Clinical Research Unit in Nursing and Midwifery, Department of Research, Women’s Wellness and Research Center, Hamad Medical Corporation, Doha, Qatar, hamad.qa; ^2^ Nursing Department, Women’s Wellness and Research Center, Hamad Medical Corporation, Doha, Qatar, hamad.qa; ^3^ Nursing Department, College of Nursing, Silliman University, Dumaguete City, Philippines, su.edu.ph; ^4^ Nursing Department, Prince Sultan Military Medical City, Riyadh, Saudi Arabia, psmmc.med.sa; ^5^ Department of Research, Women’s Wellness and Research Center, Hamad Medical Corporation, Doha, Qatar, hamad.qa; ^6^ Nursing Department, King Saud University, Diriyah, Riyadh, Saudi Arabia, ksu.edu.sa

**Keywords:** culture, equity, maternity, nursing, workforce diversity

## Abstract

**Background:**

As the world becomes increasingly multicultural, the demand for a diverse nursing workforce rises to provide equitable and high‐quality patient care. However, limited research has been conducted on these dynamics within the multicultural healthcare landscape of the Gulf region, especially in Qatar. Therefore, examining Qatar’s multicultural workforce and the diversity interaction among nurses is essential to fill this research gap.

**Aim:**

This study explored the experience of nurses and their level of interaction with a diverse workforce in a multicultural healthcare setting.

**Method:**

Sequential exploratory mixed‐methods research was conducted at a tertiary maternity facility in Doha, Qatar. In Phase I, a survey was performed with 735 nurses using the Workforce Diversity Questionnaire II, followed by focus group discussions with 10 nurses from April to June 2024.

**Results:**

The findings revealed that nurses rated highly across all domains of workforce diversity interaction. The level of interaction is influenced by age, nationality, clinical experience, diversity of patient interactions, and the length of residency in a diverse community. While there were key barriers, some factors facilitated workforce diversity interaction.

**Implications for Nursing Management:**

This study recommends the development of training programs that focus on essential competencies for nurses to enhance their performance in diverse work settings. Further investigations are also recommended to assess the impact of these competencies and training programs to patient outcomes and organizational performance.

## 1. Introduction

Workforce diversity has become a strategic priority in health sectors as teams increasingly span gender, age, nationality, language, and professional backgrounds. Aiming to create environments where every individual feels respected and supported, several organizations strategically embedded equity and inclusion. Globalization, sustained migration, and talent mobility will continue to influence diverse healthcare teams [1]. In healthcare, team diversity is linked to equitable, person‐centered care when accompanied by inclusive climates and cultural competence. Developing a diverse health care workforce that is culturally sensitive can help meet the needs of people from different nationalities. Hence, the World Health Organization (WHO) emphasized that to achieve Universal Health Coverage (UHC) and equitable care, a diverse and culturally competent workforce is essential [2]. As patient populations diversify fostering diversity, equity and inclusion remain central in addressing disparities and enabling person‐centered care [3]. A diversified healthcare team is better able to handle public health challenges and patient requirements but managing diversity is a difficult task that needs thorough preparation, transparency, and good communication [4].

In maternity care, culturally responsive teams are associated with improved trust, communication, and reduced disparities among racialized and migrant populations [5]. Evidence suggests that culturally responsive nursing can reduce barriers to access and enhance quality of care for diverse groups. However, empirical evidence on interpersonal interaction dynamics among nurses—particularly how the frequency, quality, and formality of intercultural contacts influence teamwork in maternity units—remains limited, particularly within Gulf health systems. Existing research suggests that a culturally diverse nursing workforce improves care quality for patients from diverse racial and ethnic backgrounds. Despite this, research on how nurses engage within diverse teams is limited, particularly in maternity settings.

Nurses’ perceptions of diversity and their interaction patterns are shaped by individual cultural competence, team climate, organizational routines, and broader structural inequities. Evidence suggests that cultural exposure among nurses enhances care delivery [6, 7]. As recommended, integrating cultural competence training into nursing curricula is crucial to prepare nurses for multicultural work environments [8]. In addition to personal skills, the physical setting in maternity care significantly impacts the experiences of women from various cultural backgrounds. Factors such as the ward’s arrangement, visual indicators, and environmental conditions can either facilitate or hinder effective communication, especially for underrepresented patients. [9]. These findings emphasize the need for an integrated strategy that combines workforce readiness with inclusive healthcare services and design.

Qatar has a notably diverse demographic, with expatriates accounting for approximately 88.4% of the overall population [10]. The healthcare system is significantly dependent on expatriate nurses because of the shortage of local professionals. Variations in language, educational backgrounds, and cultural standards intersect in complex settings, generating both prospects for innovation and risks of miscommunication. This particular demographic offers an excellent opportunity to investigate the diversity within the healthcare workforce. Although national initiatives have encouraged cultural understanding, there are still shortcomings in creating event‐specific skills that shape nurses’ interactions in multicultural teams [11]. This research addresses the existing gap by examining the perceptions and interactions of maternity nurses at a tertiary healthcare facility in Qatar, which has a diverse workforce. By analyzing their challenges, opportunities, and personal experiences, the research aims to inform policy development, onboarding, and team development in settings with high cultural and linguistic diversity, ultimately improving the performance of nurses and ensuring high‐quality maternity care in Qatar.

## 2. Aim

This research explored nurses’ perceptions and the degree of interaction with diverse workforce in multicultural healthcare environments.

## 3. Materials and Methods

### 3.1. Design

This sequential explanatory mixed‐methods study started with Phase I, which included cross‐sectional research aimed at assessing the extent and factors influencing nurses’ interactions within a diverse workforce. Phase II consisted of a descriptive, qualitative research investigation that explored the experiences of nurses working in a diverse work environment. The methodologies and findings from both phases were synthesized by connecting, building, and merging them through joint displays for a holistic interpretation [12, 13].

### 3.2. Quantitative Study

#### 3.2.1. Settings and Samples

A total of 735 nurses from a maternity facility in Doha, Qatar, completed the survey in Phase I. This facility is a well‐known provider of tertiary care for maternity, gynecological, and newborn services in the area. With a population of more than 3 million, Qatar has a diverse population that is primarily composed of migrants including those working in healthcare. In this study, nurses who hold a license and have at least 1 year of experience in their current clinical role were invited to participate voluntarily. Utilizing a 5% margin of error and a 95% confidence level, the minimum sample size required was 293; however, the research aimed for a participation rate of at least 60% to enhance the statistical power and validity [14].

#### 3.2.2. Instrument

The survey tool consists of three parts: Parts 1 and 2 focus on nurses’ demographic and work‐related variables. Part 3 is a modified version of Larkey’s Workforce Diversity Questionnaire‐Version II (WDQ‐II) by Beheri. Both Larkey [15] and Beheri [16] provided consent for the use of this tool, which assesses the extent of interaction among nurses in diverse workgroups. Beheri’s model includes four components: (1) inclusion/exclusion, (2) valuing differences, (3) trust, and (4) adaptation. The inclusion and exclusion capture whether someone perceives the ability or inability to genuinely connect with coworkers, indicating whether one feels socially and professionally included within the workgroup [16]. Second, valuing differences reflects how much a person recognizes and appreciates the varied beliefs, norms, and perspectives of their colleagues [16]. Third is trust which refers to their confidence in teams—the sense that others are reliable, act with integrity, and have one another’s best interests [16]. Finally, adaptation, which refers to the flexibility to adjust one’s behavior and attitude to work effectively with people from other cultural backgrounds during routine clinical work [16]. These components form the subscales of the tool, which contain 28 items rated on a 5‐point Likert scale. Total scores reflect the extent of nurses’ interaction, ranging from very poor to very high.

#### 3.2.3. Data Collection

A numerically coded master list and an online randomizer were used to select participants for the pen‐and‐paper survey. Research assistants and fellow investigators approached selected nurses, who received an information sheet and the survey tool. Completed surveys were sealed in envelopes and securely collected for analysis.

#### 3.2.4. Validity and Reliability

The WDQ‐II showed satisfactory reliability and strong validity (*α* = 0.69–0.80). [16]. A pilot test conducted at the study site, involving 30 nurses who met the inclusion criteria, proved the tool’s reliability for the intended population in Doha, Qatar (*α* = 0.78).

#### 3.2.5. Data Analysis

Quantitative data were analyzed using STATA Version 17. Descriptive statistics were used to summarize demographic and work‐related variables. Chi‐square tests were performed to assess the relationships between categorical variables. Furthermore, Spearman’s rank correlation was applied for ordinal data, particularly when the assumption of normality was not met, as indicated by the Shapiro–Wilk test.

### 3.3. Qualitative Study

#### 3.3.1. Sample

In Phase II, we followed purposive variation sampling to form two focus groups. Each was comprised of five nurses wherein one group has high interaction scores and another with low interaction scores as determined by the survey in Phase I. This sampling approach ensures a range of diverse experiences and varying perceptions allowing us to collect rich data that can illuminate or clarify the findings from Phase I [17, 18]. Instead of reaching thematic saturation, Phase II seeks to offer explanatory insights that align with the findings from Phase I of the same group [12, 13].

#### 3.3.2. Instrument

A semistructured interview guide was created to gain an in‐depth insight into workforce diversity dynamics with the nurses and to validate the findings from Phase I [19, 20]. The literature pertinent to the study was evaluated for its methodological rigor, and the results from Phase I corresponded with Beheri’s model, which informed the development of the initial semistructured interview guide [21, 22]. An expert evaluation for face validity and field testing was conducted during the pilot phase. For the expert evaluation, two subject matter experts were engaged to examine and provide their findings, particularly regarding the Phase I and the relevance of the interview questions in relation to the objectives of the study [19, 21]. The feedback from the experts was utilized to improve the open‐ended questions in the initial semistructured interview guide. Field testing was conducted by enlisting two nurses who matched the eligibility criteria of the actual study participants. In addition to relevance, the test evaluated the duration, clarity, and any other potential issues with the interview guide [23, 24]. The development and validation led to the formulation of five primary themed questions that are both engaging and exploratory, along with several supplementary questions. The creation of the interview guide was guided by the suggestions put forth by Kallio et al. [19] and Ivankova et al. [20].

#### 3.3.3. Data Collection

Focus group discussions (FGDs) were held in a confidential private meeting room at the research center of the facility. Before the sessions, written informed consent was secured. Primary questions from the interview guide and additional probing questions were asked to deepen the insights gained. Every FGD session was audio recorded and lasted between 30 and 40 minutes. The transcription function in Microsoft Word was used to transcribe every audio recording. The lead researcher served as the moderator, while a research assistant assumed the roles of observer and note‐taker. The interview preparations and process were based on the recommendations by Mack et al. [25]. All gathered information, including surveys and audio recordings, was securely stored in password‐protected digital files. The access was limited to the research team, thereby maintaining confidentiality and adherence to ethical standards.

#### 3.3.4. Data Analysis

A thematic analysis was performed following Braun and Clarke’s [26] six‐phase method: (1) becoming familiar with the data, (2) creating initial codes, (3) forming themes, (4) examining and organizing themes, (5) refining themes to ensure they align with the objectives of the study and relevant literature, and (6) presenting the findings.

### 3.4. Ethical Considerations

The research was granted ethical clearance by two Institutional Review Boards. For the academic degree, the Silliman University–University Research Ethics Committee (SUCN UREC‐2024‐196) as part of an academic requirement in the Philippines. Second approval was secured from Hamad Medical Corporation Institutional Review Board (MRC 01‐23‐905) for conducting the study at the designated site in Qatar.

### 3.5. Data Integration

The goal of the integration was to enhance both the quantitative and qualitative results, leading to richer interpretations. A sequential explanatory mixed‐methods design approach was implemented at the levels of design, methods, and interpretation. The insights gained from the quantitative data guided the collection and analysis of the qualitative data [27]. The integration process involved connecting, building, and merging [13, 28]. Interview participants for the FGDs were chosen from individuals who responded to the survey, and the interview questions were derived from the survey results. The outcomes were combined for additional analysis and interpretation as shown in joint display to illustrate the integration of both quantitative and qualitative data [29, 30]. This structured method allowed the data types to support one another, thus improving the overall understanding [31]. The STROBE checklist for cohort studies was used to ensure adherence to a rigorous methodology with enhanced validity and that the results could contribute value to the existing literature [32].

### 3.6. Trustworthiness

In line with the guidelines established by Lincoln and Guba [33], the trustworthiness of this sequential explanatory mixed‐methods research was achieved through meticulous strategies tailored for both the qualitative and quantitative components. Credibility was maintained by utilizing validated quantitative tools and performing a systematic thematic analysis of the qualitative information. The study increased coherence and depth of interpretation through data triangulation and the integration of findings from various methods. To ensure transferability, a detailed description of participant demographics and contextual conditions was provided, along with comparative insights from an additional site to support relevance in analogous situations. Dependability was assured through thorough documentation of the research framework, data collection, and analysis methods, with expert review of qualitative coding to ensure consistency and precision. Confirmability was strengthened by the use of integration matrices, which demonstrated analytic transparency, as well as reflexive journaling and audit trails to minimize researcher bias. Collectively, these strategies guaranteed the rigor, reliability, and validity of the outcomes across both aspects of the research.

## 4. Results

In line with the work of Creswell and Plano Clark [12], the results are organized by the study phase, accompanied by a cohesive synthesis. A combined visual representation was utilized to highlight the integration of both quantitative and qualitative findings [34].

### 4.1. Quantitative Findings

#### 4.1.1. Demographic and Work Profile

Table 1 presents the demographic and professional traits of the participants. The largest group of respondents was between the ages of 31 and 40 years (52.11%), and most of them were married (87.48%). A majority held a bachelor’s degree (67.89%) and were non‐Qatari nationals (98.9%). Within the non‐Qatari category, Indian nationals represented the most significant subgroup (76.5%), followed by Filipinos (14.6%) and Tunisians (3.4%). Regarding professional experience, a notable proportion of respondents had between 6 and 10 years of clinical practice (35.78%) and had lived in Qatar for a similar duration (36.73%). When asked to rate their interactions based on patients’ nationalities, respondents reported the highest levels of engagement with patients from India, the Philippines, Qatar, Egypt, and Jordan. Similarly, interactions with staff by nationality were primarily with Indian, Filipino, Qatari, Tunisian, and Egyptian colleagues.

**Table 1:** Survey participants’ demographic and work profile (*n* = 735).

Demographic variables	Frequency	Percentage (%)
Age (in years)		
20–30	39	5.31
31–40	383	52.11
41–50	233	31.70
51–65	80	10.88
Educational attainment		
Diploma	199	27.07
Bachelor’s degree	499	67.89
Master’s degree	34	4.63
Doctorate degree	3	0.41
Marital status		
Single	78	10.61
Married	643	87.48
Separated/divorced/widowed	14	1.90
Nationality		
Qatari	8	1.09
Non‐Qatari	727	98.91
Indian	**562**	**76.5**
Filipino	**107**	**14.6**
Tunisian	**25**	**3.4**
Cuban	**12**	**1.6**
Jordanian	**9**	**1.3**
Egyptian	**7**	**1.0**
Others	**5**	**0.7**
Duration of clinical experience in Qatar (years)		
1–5	164	22.31
6–10	263	35.78
11–15	99	13.47
16–20	140	19.05
21–25	41	5.58
25 above	28	3.81
Duration of living experience in Qatar (years)		
1–5	158	21.50
6–10	270	36.73
11–15	103	14.01
16–20	133	18.10
21–25	43	5.85
25 above	28	3.81

*Note:* The bold values represent the frequency and percentage of non‐Qatari nationalities.

#### 4.1.2. Extent of Diversity Interaction

Table 2 presents the degree of diversity interaction at various levels across four main areas. The majority of participants reported a high level of inclusion/exclusion (47.21%; *M* = 25.25 and SD = ±5.01), indicating a considerable ability to foster inclusivity and maintain harmonious relationships with both healthcare colleagues and patients despite cultural and national differences. A significant number of participants also expressed a strong appreciation for diversity (56.46%; *M* = 26.57 and SD = ±3.91), indicating a genuine respect for the varied backgrounds and viewpoints present in the workplace. Regarding trust, 55.82% of the respondents were within the high range (*M* = 26.29 and SD = ±3.93), showing a favorable view of their peers and the organization. The adaptation scores were the highest among all categories, with 64.22% of the participants exhibiting a high level (*M* = 27.29 and SD = ±3.66). These results suggest that nurses possess a remarkable ability to adapt and manage the dynamics of a diverse workforce and interpersonal relationships within the organization.

**Table 2:** Respondents’ interaction levels according to the four domains.

Domains	Level of workforce diversity interaction (*N*, %)					
	Very poor	Poor	Moderate	High	Very high	Mean score and description
Inclusion/Exclusion	5 (0.68)	62 (8.44)	222 (30.20)	347 (47.21)	99 (13.47)	25.25, high
Valuing differences	1 (0.14)	16 (2.18)	201 (27.35)	415 (56.46)	102 (13.88)	26.57, high
Trust	1 (0.14)	19 (2.59)	207 (28.16)	411 (55.92)	97 (13.20)	26.29, high
Adaptation	2 (0.27)	6 (0.82)	135 (18.37)	472 (64.22)	120 (16.33)	27.29, high

#### 4.1.3. Correlation Between Workforce Diversity Interaction and Respondents’ Profile

Table 3 illustrates notable relationships between age and diversity interaction domains such as inclusion/exclusion, trust, and adaptation. Nationality showed a correlation with inclusion/exclusion, the appreciation of differences, and trust. A negative correlation was identified between adaptation and the length of clinical and living experience in Qatar, indicating that shorter experiences are associated with improved adaptation. Positive correlations between adaptation and patient/workforce interactions were noted based on nationality. Although the quantitative results revealed moderate to high interactions, they fell short of completely capturing the variety of nurses’ interactions and the skills they needed. To gain a more comprehensive understanding, 10 survey participants participated in FGD which provided richer insights into the varied experiences of nurses.

**Table 3:** Chi‐square and Spearman rank correlation between the profile variable and the WDI domains.

Profile	Correlation values	Inclusion/exclusion	Valuing differences	Trust	Adaptation
Age	*x* ^2^	28.235	14.947	17.471	30.324
	*p* value	0.001^∗∗^	0.092	0.042^∗^	< 0.001^∗∗∗^

Educational attainment	*x* ^2^	10.385	2.717	5.030	4.931
	*p* value	0.109	0.843	0.540	0.553

Marital status	*x* ^2^	2.703	4.678	4.347	2.639
	*p* value	0.440	0.197	0.226	0.451

Nationality	*x* ^2^	24.699	15.318	15.360	10.514
	*p* value	< 0.001^∗∗∗^	0.018^∗^	0.018^∗^	0.105

Duration of clinical experience	*r*_*s*_	−7.884	−0.045	−0.043	−0.078
	*p* value	0.983	0.221	0.247	0.034^∗^

Duration of living experience	*r*_*s*_	−0.001	−0.058	−0.050	−0.074
	*p* value	0.968	0.116	0.176	0.046^∗^

Diversity of Patient encounter based on nationality	*r*_*s*_	0.020	0.042	0.004	0.106
	*p* value	0.584	0.254	0.910	0.004^∗∗^

Diversity of workforce encounter based on nationality	*r*_*s*_	−0.049	−0.027	−0.036	0.019
	*p* value	0.184	0.462	0.324	0.615

^∗^Significant at 0.05 level.

^∗∗^Significant at 0.01 level.

^∗∗∗^Significant at 0.001 level.

### 4.2. Qualitative Findings

The participants in the focus group discussion were female nurses, primarily between the ages of 20 and 40, who held bachelor’s degrees from various countries, including Tunisia, Indonesia, the Philippines, and India. Each of them had resided and worked in Qatar for more than 5 years, encompassing Generation X (P1 and P9), Generation Y (P2 and P4–P8), and Generation Z (P3 and P10).

In Table 4, thematic analysis revealed seven key themes: (1) establishing a supportive environment, (2) positive attitude toward diversity, (3) promoting inclusivity and equity, (4) adaptability to diversity, (5) language differences hindering staff and patient interaction, (6) role conflict between healthcare providers, and (7) generation gap between staff.

**Table 4:** An illustration table for presenting the themes, subthemes, codes, and quotations.

Themes	Subthemes	Codes	Participant quotes
1. Establishing a supportive environment	Organizational and peer support	Nurses offer support Nurses’ support as a strength Helping staff with illness Staff support each other regardless of gender Monthly unit meetings Expressing concerns Guidelines about concerns Staff improvements Use of the language bank Use of staff support resources	*“If anything happens, we all know that there is support, which is our strength… that is why we are happy as staff.”* (P5) *“I am pretty sure that the organization really supports different nationalities… or else I would not be here, right?”* (P9)
	Role modeling and mentorship	Guiding a new generation of nurses Advising new generations	*“I observe the new nurses so they will learn… I stay and advise them about the rules and policies.”* (P1)
	Fair treatment for all	Fair treatment by the organization for all Organization welcomes diversity of nationalities	*“We have a unit meeting where we can express all our issues. We can talk about work time… or any issue and try to find a solution.”* (P7)

2. Positive attitude towards diversity	Embracing diversity at work	Appreciates diversity in healthcare Working and learning from others	*“I have seen so many types of nationalities… I like diversity as you work and learn from others.” (P9)*
	Compromise of the differences	Accepts other religious practices Enhanced connectedness with staff	*“We are very diverse in terms of religion and very humble… I am Muslim… they give me time to pray… most of my colleagues were Christians.”* (P2)

3. Promoting inclusivity and equity	Celebrating diversity	Souvenirs Yearly gatherings Better understanding of others’ cultures in events	*“I am a Muslim. During Christmas time, they would give us gifts… during their vacations, they would give us souvenirs…”* (P2) *“We have this yearly gathering… understand their culture like how they are dancing… their games, their food.”* (P8)
	Encouraging integrated and diverse team collaboration	Welcoming staff Cross‐national friendships Abolishing exclusivity Work is not done exclusively by staff	*“…. No committee, no Philippine committee, no Indian committee… all of them on team… Filipino friends with Tunisian and Indian with Tunisian.”* (P1)

4. Adaptability to diversity	Adjusting to cultural differences in the workplace	Social openness Likes new experiences with diversity Learning English Improved communication skills	*“I like diversity. I started eating different kinds of foods from other countries… we started going outside of work to visit restaurants.”* (P8)
	Generational adaptation and team compromise	Adapting work pacing Compromise Accepting others’ approach Experience enhances adaptation Suggesting innovations	*“The younger nurses want fast and get a result… mature nurses want to be slow but accurate… I do believe we compromised.”* (P9)

5. Language as barrier to nurses to staff or patient interaction	Language barriers affecting patient care	Language difference Staff cannot speak English fluently	*“Arabic patients refuse certain nurses because they do not understand each other… this affects patient assignment.”* (P7)
		Need for explanation Challenges in seeking representation Asking for help because of language concerns	*“Explanations are more important to avoid miscommunication… we ask the patient if they understand.”* (P6)
	Miscommunication in staff collaboration	Exclusive language use Comprehension of the native/national language	*“I can see nurses together speaking in Malayalam… I cannot understand what is happening inside.”* (P8)
		Staff cannot help because of language concerns. Dialects create division	*“… When they are communicating with their dialects, it becomes a challenge for others.”* (P7)

6. Role conflict between healthcare providers	Miscommunication and unclear role expectations	Differences in role awareness Misunderstanding about requests/orders	*“…. Sometimes, they understand it differently… I do not know what they are going to say… they will say no or say it in an insulting way.”* (P2)
	Cultural and gender‐driven role expectations	Patient gender preference Preferred female over male staff Challenges in seeking gender representation	*“Patients will ask… for a female doctor… we have to call female doctors and convince them to come even if they are not on duty.”* (P8)

7. Generation differences	Age diversity and generation gap	Generational differences in responsiveness Older nurses’ feelings of exclusion Younger nurses are overlooking older nurses Doubts about new nurses’ proficiency	*“Still, they need us, but… they are not accepting this point. Some are accepting, but this is the challenge.”* (P1) *“The younger generations work fast in their own pace with technology. But then you feel left out already because they work fast when they do this type of procedure. They do things fast.”* (P9)
	Difference in work attitudes across generations	Generational difference in work approach Disagreement between generational work groups	*“They would answer why they are called because they are not on call… but we still have like 60 plus working bedside without any questions.”* (P5)

#### 4.2.1. Theme 1: Establishing a Supportive Environment

Participants consistently emphasized the significance of a supportive workplace culture in fostering trust, inclusivity, and team stability. They noted that having access to guidance, open communication, and equitable treatment made them feel valued and secure. Therefore, nurturing support enhances diverse interactions, fostering respect, collaboration, and loyalty, which are crucial for nurse satisfaction in a diverse work environment.

##### 4.2.1.1. Subtheme 1.1: Organizational and Peer Support

The nurses appreciated the support from their fellow staff and managers, both at the unit and departmental levels, as well as at the organizational level. They viewed this support as powerful and nonexclusive. Nurses shared:

*“If anything happens, we all know that there is support, which is our strength… that is why we are happy as staff.” (P5)*

*“I am pretty sure that the organization really supports different nationalities. All nurses, or else I would not be here, right?” (P9)*

##### 4.2.1.2. Subtheme 1.2: Role Modeling and Mentorship

The support was perceived as a type of mentorship, and measures were taken to avoid any potential conflicts of interest. Typically, senior nurses engage actively in enforcing compliance with regulations while mentoring junior staff. Nurses described:

*“I observed the new nurses so they would learn. I ensure that nurses do not create a significant problem. So, I stay and advise them about the rules and policies. I remind them that we have a policy, like guidelines.”* (P1)

##### 4.2.1.3. Subtheme 1.3: Fair Treatment for all

Regular team meetings served as another method for establishing support within the organization. *“We have a unit meeting where we can express all our issues. We can talk about work time, patient problems, violence, or any issue and try to find a solution.”* (P7)

Such organizational support mechanisms were seen as critical to fostering collaboration, loyalty, and harmony within the diverse nursing workforce.

#### 4.2.2. Theme 2: Positive Attitude Toward Diversity

Participants highlighted that a friendly and inclusive attitude toward cultural, racial, and religious diversity is crucial for fostering inclusion and enhancing team dynamics. Nurses emphasized the importance of respect and the valuable learning experiences that diversity can offer.

##### 4.2.2.1. Subtheme 1.1: Embracing Diversity at Work

This attitude fostered mutual learning and respect among staff members from diverse backgrounds, ultimately contributing to a cohesive and collaborative environment. The nurse explained:

*“I have seen so many nationalities that I have been working not only from here but from the previous. I like diversity, as you work and learn from others.”* (P9)

##### 4.2.2.2. Subtheme 2.2: Compromise of the Differences

This respectful and appreciative stance toward diversity was not only a source of personal comfort but also instrumental in fostering inclusive collaboration and improving the quality of nursing care delivery. Another participant reflected on the religious inclusivity in the unit:

*“For me, I also notice religion. We are a diverse group in terms of religion, and also very respectful. Moreover, I am a Muslim. However, if I want to pray, they will give me time to pray especially that most of my colleagues are Christians.”* (P2)

#### 4.2.3. Theme 3: Promoting Inclusivity and Equity

The third theme focuses on enhancing inclusivity and equity by fostering fairness, respect, and cultural sensitivity in the workplace. These results underscore the importance of deliberate initiatives aimed at promoting equity and inclusivity in effectively integrating a diverse workforce, building unity, and enhancing the standard of care provided.

##### 4.2.3.1. Subtheme 3.1: Celebrating Diversity

Yearly social gatherings were recognized as a way to promote unity and cultural exchange. For example, the nurses emphasized the importance of cultural sensitivity and respect for varying religious beliefs. *“I am a Muslim. During Christmas time, they would give us gifts. Yeah, it is really sweet. Even during their vacations, they would give us souvenirs from different countries.”* (P2)

*“We have this yearly gathering for the unit’s party. All are invited. This activity can relieve stress. We get the opportunity to interact with each other, understand their culture, like how they dance, their games, their food…”* (P8)

##### 4.2.3.2. Subtheme 3.2: Encouraging Integrated and Diverse Team Collaboration

Participants spoke about how inclusive practices facilitated connections across differences in nationality, age, religion, and educational background. The nurses highlighted the benefits of removing segregation in the workplace and promoting collaboration among diverse groups.

*“If you have any segregation in your unit, you have to destroy it. No committee for the Philippines, no Indian committee, no Tunisian committee; it should be all of them in one team. That is why… we find Filipino friends with Tunisians and Indians with Tunisians.”* (P1)

#### 4.2.4. Theme 4: Adaptability to Diversity

This theme examines how nurses adjust and evolve in response to the diversity in their workplaces. The ability to adapt has been demonstrated to enhance collaboration, inclusivity, and the delivery of culturally competent care. Nurses noted that adjusting to cultural and generational differences enabled them to establish stronger relationships with their colleagues and enhanced their ability to care for patients from diverse backgrounds. This theme showcases how small, everyday instances of cultural exchange served as connections between individuals of different nationalities. These interactions were not merely social but also helped create a sense of belonging and connection across diverse groups.

##### 4.2.4.1. Subtheme 4.1: Adjusting to Cultural Differences in the Workplace

Nurses reported that embracing and adapting to the cultural norms of others improved their professional and personal interactions. Exposure and sharing cultural practices served as a starting point for connection and learning. The nurse described:

*“I like diversity. I started eating various foods from different countries. This experience helps me connect with other nurses from other countries. So, we started going outside of work to visit restaurants.”* (P8)

##### 4.2.4.2. Subtheme 4.2: Generational Adaptation and Team Compromise

Participants shared both obstacles and strategies encountered while collaborating with colleagues of varying ages and experience levels. Instead of letting these differences create tension, they adjusted to different approaches to work, emphasizing shared objectives for patient safety. The nurse said:

*“The younger nurses want fast results. When it comes to mature nurses, they want to be slow but accurate, so they want to know. However, I do believe that is really how each group will adapt and adjust for the benefit of the patient and compromise. I do believe we compromised.”* (P9)

#### 4.2.5. Theme 5: Language Differences Hindering Staff and Patient Interaction

Communication challenges became a significant obstacle in delivering adequate care and maintaining team unity. In a diverse environment, difficulties in communication—whether with patients or among team members—interrupt the workflow, lead to misunderstandings, and increase the risk of errors.

##### 4.2.5.1. Subtheme 5.1: Language Barriers Affecting Patient Care

The issue of diversity goes beyond just interactions among staff. Nurses emphasized the importance of ensuring patients understand and proactively seek translation assistance or more straightforward explanations when needed. Nurses indicated that language barriers hindered both patient assignments and emergency responses. The nurse shared:

*“Arabic patients refuse certain nurses because they do not understand each other due to language. This affects patient assignment and focus of the staff.”* (P7)

“*Explanations are more important to avoid miscommunication. Expressing something, but they [patients] will understand it differently. So, we ask the patient if they understand, or if they want more explanation, then we ask for help from others.”* (P6)

##### 4.2.5.2. Subtheme 5.2: Miscommunication in Staff Collaboration

The presence of multiple languages within units also led to exclusion and misunderstandings among nurses from various national backgrounds. Variations in language can seriously affect both the quality of care provided to patients and the effectiveness of collaboration among teams in multicultural environments. Misunderstandings can lead to patient dissatisfaction, disjointed teamwork, and a decline in staff confidence. To address this issue, it is crucial to establish structured language support, provide training in cross‐cultural communication, and develop policies that foster inclusive discussions within diverse healthcare teams.

Nurses shared:

*“I can see Indians together speaking in Malayalam [Indian ethnic language] and cannot understand. They are running to the emergency room, and I cannot understand what is happening inside. Then later I learned that they are asking each other for help.”* (P8)

*“I am working in Gynecology units, and we are from different nationalities: Tunisians, Filipinos, Indians, so when they are communicating with their dialects, it becomes a challenge for others.”* (P7)

#### 4.2.6. Theme 6: Role Conflict Between Healthcare Providers

Role conflict occurs when the responsibilities, expectations, and professional boundaries among healthcare providers are unclear or misinterpreted, leading to confusion and potential issues. This challenge hinders effective collaboration, especially in diverse environments where cultural practices and hierarchical expectations differ. Misunderstandings regarding roles and authority can create friction, delays in patient care, and dissatisfaction among team members. Nurses often find themselves in a challenging position, having to balance cultural sensitivity with operational constraints, which underscores a role conflict that goes beyond individual responsibilities into the realm of interprofessional alignment. Role conflict signifies more than just discrepancies in tasks; it reveals underlying issues related to hierarchy, cultural negotiation, and the adaptability of institutions.

##### 4.2.6.1. Subtheme 6.1: Miscommunication and Unclear Role Expectation

Nurses often encounter ambiguity when their roles overlap with those of other professionals, such as physicians. Requests for assistance or collaboration may be received with resistance, particularly if the communication is perceived as overstepping boundaries or intrusive. This experience promotes reluctance, worry, and a feeling of professional disempowerment. The nurse shared:

*“I think one of the challenges may be miscommunication. Sometimes, they understand it differently, even though they mean it differently. Perhaps you could ask a doctor for an order, as it is their role, and then we’ll ask politely in front of them. I do not know what they are going to say. Although they may say no, we are busy right now, or they might say so. In an insulting way.”* (P2)

This narrative demonstrates how role‐related expectations and the commencement of therapeutic decisions can be misinterpreted or greeted with hostility, resulting in emotional distress and divided teamwork.

##### 4.2.6.2. Subtheme 6.2: Cultural and Gender‐Driven Role Expectations

Cultural expectations regarding gender and healthcare provision can lead to conflicts, especially when the established protocols of institutions conflict with patient wishes. Nurses often find themselves caught between honoring the cultural needs of patients and adhering to strict professional guidelines, which can result in stress and obstacles in delivering care. The nurse said:

*“Patients will ask from the front triage for a female doctor. For them, it is unacceptable to be seen by a male. However, it will be a challenge because we have to call female doctors and convince them to come even if they are not on duty.”* (P8)

#### 4.2.7. Theme 7: Generation Differences

Variations in generational perspectives among nurses affect their views on responsibility, communication methods, and flexibility. Senior nurses frequently expressed concerns about how younger nurses approach their roles and dedication. Customized strategies are crucial for bridging the generational boundaries in nursing, promoting collaboration, and maintaining a cohesive healthcare environment.

##### 4.2.7.1. Subtheme 7.1: Age Diversity and Generation Gap

Experienced nurses often express concerns about the younger generation’s attitude toward responsibility and dedication. Nurses from this cohort appear less eager to embrace new roles and show hesitance in adopting new technologies. As many face the challenges of aging and health concerns, their capacity to engage in new tasks differs significantly from that of the younger nurses. Nurses shared.

*“When we call for new nurses, they would answer why they are called because they are not on call or duty. However, still, we have 60-year-olds working bedside without any questions; they will come directly.”* (P5)

*“The younger generations work fast in their own pace with technology. But then you feel left out already because they work fast when they do this type of procedure. They do things fast.”* (P9)

##### 4.2.7.2. Subtheme 7.2: Difference in Work Attitudes Across Generations

Generational differences among nurses can influence perceptions of responsibility, communication style, and adaptability.

*“For one generation, but it does not mean the next generation is good or bad; it depends on the situation. Still, they [other generation nurses] need us, but for some, this point is not being accepted. Some are accepting, but this is the challenge.”* (P1)

### 4.3. Integration Process

**FIGURE 1:**
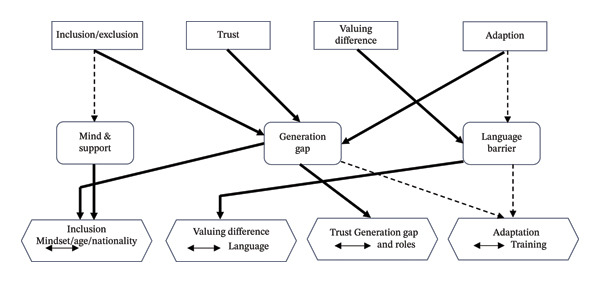
Joint display of the quantitative and qualitative findings. Legend: rectangle: “survey domain;” rounded rectangle: “key theme;” hexagon: “practice inference;” solid, thick arrow: strong or primary relationship. Dashed Arrow: secondary or supporting relationship.

Data integration was completed by utilizing a joint display and narrative weaving in accordance with the guidelines suggested by Fetters and colleagues [13]. This thematic alignment was grounded in the significance of findings from both quantitative and qualitative datasets. The integration highlighted obstacles and enablers to diversity interaction, as illustrated in Table 5 and Figure 1. Several themes identified in Phase II emerged as enablers of increased diversity and interaction, including a supportive atmosphere, a positive mindset, inclusiveness, equity, and adaptability. The other themes, numbered 5–7, were identified as barriers, including language differences, role conflicts, and generational gaps.

**Table 5:** Joint display of the quantitative and qualitative findings.

Quantitative findings	Qualitative findings	Inferences
• Moderate (30%) to high (47%) inclusion/exclusion • Correlated with age and nationality	• Generation gap • Promoting inclusivity and equity • Establishing a supportive environment	• Promoting inclusion facilitates interaction among diverse groups, whereas age diversity can be a barrier.

• Moderate (27.35%) to high (56.46%) valuing difference • Correlated with nationality	• Positive attitude to diversity • Language as a barrier to nurses’ interaction with staff or patients’ interaction	• Language from different nationalities can be a barrier to valuing differences, whereas having a positive mindset can facilitate diverse interactions.

• Moderate (28.16%) to high (55.92%) trust • Correlated with age and nationality	• Generation gap • Role conflict between healthcare providers	• Trust remains a concern with generational and role conflicts. Thus, both are barriers to trust.

• Moderate (18.37%) to high (64.22%) to adaptation • Correlated with age and the diverse encounters of patients based on their nationalities • Negative correlation with the duration of clinical and living experience	• Generation gap • Language as a barrier to nurses’ interaction with staff or patients’ interaction • Adaptability to diversity	• Barriers such as the generation gap and language concerns are complemented by the nurses’ ability to adapt to diversity.

Figure 1 illustrates the joint display analysis matrix found in Table 5. The matrix that combines both quantitative and qualitative findings offers insights that can enhance the understanding of workforce diversity interactions within healthcare environments. In Domains 1 and 2, nurses received high scores for inclusion/exclusion and appreciation of diversity. In Domain 1, a strong emphasis on inclusion reflects respectful interactions and engagement with a variety of work groups. Qualitative interviews uncovered methods to promote inclusivity and reduce exclusivity. Notably, younger nurses from India and the Philippines demonstrated greater levels of inclusion, along with those who had attained higher educational qualifications. Nevertheless, issues such as feelings of exclusion among older nurses and language barriers may account for the low diversity interaction scores. For Domain 2, nurses demonstrated a high value in valuing differences, which they viewed positively as helping them grow professionally and become more culturally competent. However, nurses also view that the differences, when not viewed positively, can be a barrier to working with diverse teams. However, sharing knowledge and expertise can resolve these problems. Other challenges in valuing differences include generational gaps and concerns about language proficiency. For instance, new nurses may be unwilling to extend their working hours and days. In contrast, experienced nurses may be resistant to new ideas or struggle to keep up with technological advancements. In Domain 3, results showed high trust levels, indicating that, despite diversity, nurses support one another as a team. They appreciate their organization for the freedom to raise their perspectives. Nurses are not selective, but they face challenges such as patients preferring female healthcare providers, which can affect decision‐making and communication. In terms of nationality, both Filipino and Indian nurses foster trust compared with diverse groups. Lastly, the survey results indicate that nurses scored high adaptation levels, suggesting they are embracing and can work with a diverse workforce. They learn to work with diverse work groups and acquire and compromise with their fellow team members’ cultural practices. In addition, both age diversity and language matter in terms of adaptability. Older nurses mentor younger nurses, allowing them to work at their own pace while reminding them of organizational protocols and best practices. However, language barriers can hinder adaptation, as nurses must find proficient communicators for patients or colleagues. Inferences based on these findings suggested that although promoting inclusion supports interaction, both language and age diversity could hinder interaction. Generational and role conflicts challenged trust, but adaptability could help resolve these challenges.

## 5. Discussion

This study findings indicate that organizational, environmental, and professional supports—particularly psychologically safe unit climates, structured mentorship, and regular team discussions—correlate with stronger inclusion, higher perceived trust, and greater teamwork within a diverse maternity nursing workforce. A psychologically safe work environment was associated with higher trust, increased job satisfaction, and reduced turnover intentions, consistent with previous findings that link organizational support to staff morale and retention [35]. Structured mentorship, which pairs experienced and novice nurses, promotes acculturation to policies, communication standards, and team expectations. This approach not only mitigates workplace issues but also cultivate a sense of community, which is essential for retaining staff nurses [36]. Furthermore, unit meetings encouraged transparent communication, enhancing respect and teamwork among diverse groups. In general, organizational and professional foster a sense of belonging and facilitate effective teamwork in multicultural care settings.

A constructive perspective on diversity fosters inclusivity, enhances teamwork, and enhances the standard of patient care, as illustrated by the research findings. Nurses emphasized the significance of cultural and religious diversity as opportunities for personal and professional growth, while improving their diversity competencies which corresponded with increased perceptions of inclusiveness and respect. This recognition of diversity fostered mutual respect and a greater sense of belonging within teams. Interestingly, perception varied by nationality with different groups indicating increased levels of inclusion and trust. This positive attitude may stem from experiences in multicultural care or access, training in cultural competence, and language‐concordant networks [37]. While early‐career nurses demonstrated a strong commitment to inclusivity, yet reported more difficulties with adaptability, which is indicative of their novice status and the development of role confidence and emotion regulation in early practice [38]. These insights underscore the need for ongoing cultural competence training, particularly for individuals new to the country. Additionally, promoting inclusiveness and equity through equitable treatment, cultural awareness, and respect for diversity is essential for cultivating a unified workplace. Nurses noted that offering equal opportunities, regardless of nationality, religion, or background, minimizes exclusion and fosters a sense of belonging. The incorporation of cultural competence education into nursing programs in various countries prepares nurses to collaborate and engage effectively with diverse teams [37]. Complementary activities, including inclusive festivities and team‐building events, were viewed as effective methods to foster cultural understanding and enhance team unity. This finding corresponds with existing research, which confirms that inclusivity not only enhances morale but also improves the quality of care and overall team performance [7]. Promoting collaboration rather than division and appreciating diversity are crucial for fostering equity in multicultural healthcare environments. Flexibility is essential for promoting cooperation, inclusivity, and culturally sensitive care among diverse healthcare teams. Nurses participating in the research indicated that accommodating various cultural backgrounds and work practices improves teamwork and fosters a more supportive atmosphere. This adaptability also significantly helps to bridge generational divides within teams. Meanwhile, while younger nurses are generally more inclusive, they may encounter difficulties in adaptability due to their relative lack of experience and emotional maturity. Conversely, nurses in their 30s and 40s show increased adaptability, likely due to numerous professional development opportunities and leadership roles they have undertaken. [39]. Furthermore, achieving a higher level of education is correlated with greater openness and cultural awareness although frequent interaction with diverse groups appears to have a similarly significant impact [40]. Clinical experience yields varied impacts, as both inexperienced and experienced nurses demonstrate flexibility in diverse situations [6, 41]. Personal factors, including marital status, impact adaptability, with nurses who are widowed indicating reduced levels, possibly as a result of burnout. In Qatar’s context, hiring nurses from various nations not only meets staffing requirements but also improves cultural sensitivity in the delivery of patient care. Barriers to successful collaboration in diverse healthcare environments include language issues, conflicts over roles, and generational differences. Language difficulties can impede patient care and teamwork when nurses and patients do not share a common language, and the use of native dialects might result in miscommunication or feelings of exclusion [41, 42]. Research conducted in Canada, the UAE, and Bahrain highlights these issues, indicating that dependence on translators or casual communication frequently hinders collaboration [42].

Moreover, role conflicts, especially between physicians and nurses, often arise from poor communication and ambiguous responsibilities, underscoring the importance of clearly defined roles and mutual respect [43]. Generational differences further complicate the dynamics, as younger nurses may be more open to change and empathy, while older nurses sometimes resist new practices [44]. These factors underscore the importance of structured strategies that address communication, clarify roles, and bridge generational gaps to support a cohesive and inclusive work environment.

Building on these findings, another key finding from this sequential explanatory mixed‐methods study highlights the importance of developing organized core competencies for practical training and workforce diversity management in maternal care. These interventions could increase nurses’ interaction levels to a very high level. These suggested competencies, based on the Knowledge, Skills, and Attitude (KSA) model, can serve as a foundation for developing a unified and high‐performing healthcare workforce. First, building an inclusive culture in which nurses feel appreciated and encouraged leads to better teamwork, mentorship, and engagement. Such surroundings not only promote collaboration but also improve patient outcomes. Second, cultural competency, as a core component, can enhance effective communication and provide culturally responsive care that meets the diverse needs of both patients and employees [45].

Furthermore, a favorable attitude toward diversity fosters team cohesion and happiness, primarily when nurses’ cultural beliefs are acknowledged and respected. This third competency promotes inclusive decision‐making and strengthens interprofessional collaboration. [45]. Conflict resolution is another skill that develops after negotiating cultural misconceptions and diverse perspectives. Adapting to conflict management tactics improves teamwork, creativity, and ethical reasoning. The fourth skill is trust among teams, which, when combined with guided reflection, can contribute to a psychologically safe atmosphere in which diversity‐related issues can be handled [46]^.^ The fifth capability is adaptation, which entails accepting multiple perspectives and adapting to changing practices. In maternity care, adaptive nurses play a crucial role in providing inclusive care and enhancing service quality. When combined with reflective practice, adaptability decreases bias, promotes professional development, and improves communication and satisfaction [47]^.^ Ultimately, the importance of self‐reflection in acknowledging personal biases and prejudices is crucial. Reflective nurses are better equipped to adjust their behavior, leading to more respectful teamwork and improved care. As a result, these competencies serve as an essential foundation for future training, development, and research aimed at increasing workforce diversity interaction in maternity care.

### 5.1. Limitations of the Study

Self‐reporting and single‐site sampling limit generalizability; triangulation and contextual details were used to augment credibility and transferability. However, the study’s strength lies in its sequential explanatory research strategy, which complements qualitative research findings with quantitative findings. Furthermore, the original insights it provides into nurses’ relationships with diversity in a multicultural Arab region, notably the obstacles and opportunities, present necessary paths for nursing education and future nursing and social science research.

## 6. Conclusion

The study emphasizes the importance of diversity, inclusion, and teamwork actionable in maternity care. Results show that supportive environments, cultural competence, and adaptability improve teamwork and patient care quality. Specific tactics are required to overcome barriers such as language and generational disparities. Integrating qualitative and quantitative insights leads to a more comprehensive knowledge, which supports policy formulation and education to improve diverse healthcare teams, particularly among nurses in Qatar’s dynamic maternal healthcare settings. Lastly, by institutionalizing these approaches and assessing inclusion‐sensitive indicators, health systems can enhance worker unity and patient experience while establishing a foundation for thorough impact evaluation.

## 7. Implications and Recommendations

The findings have significant implications for nursing education, practice, policy, and future studies concerning the diverse workforce in healthcare. In the realm of nursing education, it is vital to incorporate the identified competencies into nursing curricula to adequately prepare nurses for thriving in multicultural healthcare environments. In nursing practice, hospital administrators can strategically create a roadmap that sets directions for building an inclusive healthcare working environment and nurturing trust with the stakeholders. The roadmap also provides milestones in developing nurses to become competent in working with their diverse healthcare workforce and clientele. Aside from cultural competence, conflict resolution, developing trust, maintaining a positive attitude toward diversity, and adaptability are recommended for the ongoing professional development programs. Implementing structured mentorship and peer support systems that promote inclusivity and equity can significantly impact nursing performance and patient care outcomes, underscoring the importance of the proposed nursing competencies. In terms of research, the researchers aim to create a tool that assesses this emerging nursing competency in relation to diversity. Future studies should investigate how integrating diversity competency into nursing curricula affects patient outcomes, with a particular focus on addressing health disparities and ensuring universal healthcare coverage.

## Author Contributions

Conceptualization: John Paul Ben T. Silang, Evalyn Abalos, Barbara Lyn A. Galvez, Theresa Guino‐o, David Hali de Jesus, Hazel F. Adalin, Norisk M. Adalin, Rana Aatif Salim Ibrahem, and Jameela Syed Roshan Nuddin.

Investigation: John Paul Ben T. Silang, Evalyn Abalos, Barbara Lyn A. Galvez, Theresa Guino‐o, and David Hali de Jesus.

Methodology: John Paul Ben T. Silang, Evalyn Abalos, Barbara Lyn A. Galvez, Theresa Guino‐o, David Hali de Jesus, and Norisk M. Adalin.

Data collection: John Paul Ben T. Silang, RASI pertaining to Rana Aatif Salim Ibrahim, Jameela Syed Roshan Nuddin, David Hali de Jesus, and Rana Aatif Salim Ibrahem.

Data curation: John Paul Ben T. Silang, Evalyn Abalos, Hazel F. Adalin, Norisk M. Adalin, Rana Aatif Salim Ibrahem, and Jameela Syed Roshan Nuddin.

Formal analysis: John Paul Ben T. Silang, Evalyn Abalos, Barbara Lyn A. Galvez, Theresa Guino‐o, David Hali de Jesus, and Norisk M. Adalin.

Project administration: John Paul Ben T. Silang, Evalyn Abalos, Barbara Lyn A. Galvez, Theresa Guino‐o, David Hali de Jesus, HA, Norisk M. Adalin, Rana Aatif Salim Ibrahem, and Jameela Syed Roshan Nuddin.

Visualization: John Paul Ben T. Silang, Evalyn Abalos, Barbara Lyn A. Galvez, Theresa Guino‐o, David Hali de Jesus, and Norisk M. Adalin.

Writing–original draft: John Paul Ben T. Silang, Evalyn Abalos, Barbara Lyn A. Galvez, Theresa Guino‐o, and David Hali de Jesus.

Validation: John Paul Ben T. Silang, Evalyn Abalos, Barbara Lyn A. Galvez, Theresa Guino‐o, David Hali de Jesus, and Norisk M. Adalin.

Supervision: John Paul Ben T. Silang, Evalyn Abalos, Barbara Lyn A. Galvez, Theresa Guino‐o, and David Hali de Jesus.

Writing–review and editing: John Paul Ben T. Silang, Evalyn Abalos, Barbara Lyn A. Galvez, Theresa Guino‐o, David Hali de Jesus, Hazel F. Adalin, Norisk M. Adalin, Rana Aatif Salim Ibrahem, and Jameela Syed Roshan Nuddin.

## Funding

The publication of this research article (MRC 01‐23‐905) was funded by the Medical Research Center of Hamad Medical Coporation, Doha, Qatar.

## Disclosure

All authors have read and approved the final draft that was submitted for publication.

## Conflicts of Interest

The authors declare no conflicts of interest.

## Data Availability

The data used to support the findings of this study are available from the corresponding author upon reasonable request.
